# TLR4-interacting SPA4 peptide improves host defense and alleviates tissue injury in a mouse model of *Pseudomonas aeruginosa* lung infection

**DOI:** 10.1371/journal.pone.0210979

**Published:** 2019-01-28

**Authors:** Shanjana Awasthi, Bhupinder Singh, Vijay Ramani, Jun Xie, Stanley Kosanke

**Affiliations:** 1 Department of Pharmaceutical Sciences, University of Oklahoma Health Sciences Center (OUHSC), Oklahoma City, Oklahoma, United States of America; 2 Department of Pathology, OUHSC, Oklahoma City, Oklahoma, United States of America; Louisiana State University, UNITED STATES

## Abstract

Interaction between surfactant protein-A (SP-A) and toll-like receptor (TLR)4 plays a critical role in host defense. In this work, we studied the host defense function of SPA4 peptide (amino acids GDFRYSDGTPVNYTNWYRGE), derived from the TLR4-interacting region of SP-A, against *Pseudomonas aeruginosa*. We determined the binding of SPA4 peptide to live bacteria, and its direct antibacterial activity against *P*. *aeruginosa*. Pro-phagocytic and anti-inflammatory effects were investigated in JAWS II dendritic cells and primary alveolar macrophages. The biological relevance of SPA4 peptide was evaluated in a mouse model of acute lung infection induced by intratracheal challenge with *P*. *aeruginosa*. Our results demonstrate that the SPA4 peptide does not interact with or kill *P*. *aeruginosa* when cultured outside the host. The SPA4 peptide treatment induces the uptake and localization of bacteria in the phagolysosomes of immune cells. At the same time, the secreted amounts of TNF-α are significantly reduced in cell-free supernatants of SPA4 peptide-treated cells. In cells overexpressing TLR4, the TLR4-induced phagocytic response is maintained, but the levels of TLR4-stimulated TNF-α are reduced. Furthermore, our results demonstrate that the therapeutic administration of SPA4 peptide reduces bacterial burden, inflammatory cytokines and chemokines, intracellular signaling, and lactate levels, and alleviates lung edema and tissue damage in *P*. *aeruginosa-*infected mice. Together, our results suggest that the treatment with SPA4 peptide can help control the bacterial burden, inflammation, and tissue injury in a *P*. *aeruginosa* lung infection model.

## Introduction

Antimicrobial resistance and the acquisition of new virulence traits have contributed to a worldwide increase in the incidence of infections and associated morbidity and mortality.[1–3] Therefore, new therapeutic approaches are urgently needed to control difficult-to-treat infections. One way of addressing this need is to harness natural immune defenses of the host to develop therapeutic entities.

Secreted surfactant protein-A (SP-A) in lung alveoli helps reduce surface tension and maintain normal lung function, and contributes to host defense. SP-A utilizes different mechanisms and facilitates clearance of respiratory pathogens. Specifically, it reduces microbial growth by increasing the membrane permeability of Gram-negative bacteria,[4–7] and fungal pathogen,[8] and stimulates the pathogen recognition, clearance, and immune responses of phagocytes through its interaction with calreticulin/CD91, signal regulatory protein (SIRP)α, Toll-like receptors (TLRs), and SP-R210.[4, 9, 10] Secreted levels of SP-A are, however, reduced during lung infection and inflammatory conditions.[11, 12] Replenishing SP-A in such scenarios could aid in the elimination of pathogens. Despite an understanding of the host defense role, the use of SP-A for therapeutic purposes has been difficult due to its large size, amenability to degradation, and undesirable pro-inflammatory effects of the N-terminal region of SP-A, through its binding to calreticulin/CD91.[13]

We have focused on investigating the host defense function of SP-A through its interaction with Toll-like receptor 4 (TLR4).[14] TLR4 is expressed by immune cells and some non-immune cells, and its expression is further increased during infection and inflammation.[15] While TLR4 recognizes pathogens, stimulates phagocytosis, and coordinates innate and adaptive immunity, activation of TLR4 leads to exaggerated inflammation and tissue injury through intracellular myeloid differentiation primary response (MYD88) and Toll/interleukin-1 receptor (TIR) domain-containing adaptor inducing interferon-β (TRIF) signaling pathways.[16, 17]

We previously reported that purified native lung SP-A interacts with TLR4 and promotes bacterial phagocytosis, yet suppresses the inflammatory cytokine response.[14] These findings led us to examine whether short TLR4-interacting regions of SP-A can maintain some of the host defense functions of SP-A. Using computational molecular modeling and docking, we identified TLR4-interacting regions of SP-A.[18] Our work revealed that the lead SPA4 peptide (amino acid sequence: GDFRYSDGTPVNYTNWYRGE) binds to recombinant TLR4-myeloid differentiation protein 2 (MD2). MD2 is an adaptor molecule, and is involved in recognition and binding to the TLR4-ligand and forming a complex with TLR4 on cell surface for stimulation of downstream signaling. Furthermore, our results demonstrated that the SPA4 peptide suppresses the TLR4-MYD88-induced inflammatory response against Gram-negative bacterial lipopolysaccharide (LPS; a potent ligand of TLR4) in cell systems and in a mouse model.[18–20]

In the present study, we examined whether SPA4 peptide affects the TLR4-induced phagocytic and inflammatory response against *Pseudomonas aeruginosa*, an LPS-expressing Gram-negative bacteria, and evaluated its biological affects *in vitro* and in a mouse model of *P*. *aeruginosa*-induced lung infection and inflammation. Our results demonstrate that the SPA4 peptide treatment induces uptake of *P*. *aeruginosa*, but suppresses cytokine response at a cellular level leading to reduced bacterial burden and inflammation, and alleviation of lung injury in mice.

## Materials and methods

### SPA4 peptide

The SPA4 peptide (amino acid sequence: GDFRYSDGTPVNYTNWYRGE; Genscript, Piscataway, NJ) was included in this study. In addition, fluorescein isothiocyanate (FITC)-conjugated SPA4 peptide was used in some assays. The purity of each batch of the peptides was confirmed by mass spectroscopy and high-performance liquid chromatography (HPLC). The peptides were reconstituted in endotoxin-free water. Batch preparations of the peptide solutions were tested for endotoxin contamination by Limulus amebocyte lysate assay (Charles River, Charleston, SC). Heat-inactivated SPA4 peptide was prepared by heating the SPA4 peptide solution at 99°C for 4 h.

### Animals

We included C57BL6/J mice (5-to-6-week-old females; Jackson Animal Laboratory, Bar Harbor, ME) for harvesting the primary alveolar macrophages and for evaluating the biological activity of SPA4 peptide in a model of *P*. *aeruginosa* lung infection. All mice were acclimatized for at least one week prior to performing experiments, and were randomly allocated to experimental groups. Mice were given food and water *ad libitum*. Mice were anesthetized using isoflurane or ketamine/xylazine anesthesia. Animal protocols were approved by the OUHSC Institutional Animal Care and Use Committee (Protocol numbers 14-054-HI, 12-105-HI, and 17-081-CHR).

### Cell culture and maintenance

Murine-derived JAWS II dendritic cells (ATCC, Manassas, VA) were maintained as described earlier.[20] Primary alveolar macrophages were harvested from C57BL6/J mice as per the published method.[21, 22] The viability and morphology of cells were monitored using the trypan blue dye exclusion method and Wright-Giemsa staining, respectively.[23]

### Pseudomonas aeruginosa

*P*. *aeruginosa* PAO1[24] and green fluorescent protein (GFP)-expressing *P*. *aeruginosa* 8830[25] strains (obtained from Dr. William McShan, Department of Pharmaceutical Sciences, OUHSC, OK) were maintained in tryptic soy broth or agar medium. The bacterial cultures were characterized for biochemical characteristics at the Microbiology lab, University of Oklahoma Medical Center, Oklahoma City. As expected, *P*. *aeruginosa* colonies were positive for both catalase and oxidase enzymes (BD Biosciences, San Jose, CA), and maintained Gram-negative staining and colony and growth characteristics throughout the study.

### Predictions about the antimicrobial regions within SPA4 peptide

The amino acid sequence of SPA4 peptide was screened for an antimicrobial domain using the freely available Collection of Anti-Microbial Peptides (CAMPR3) database,[26] Antimicrobial Sequence Scanning System (AMPA) algorithm,[27] Antimicrobial Database (APD3),[28] and Web-based Prediction of Aggregation-prone Segments (AGGRESCAN) program.[29] The CAMPR3 database is composed of sequences, structures, and family-specific signatures of prokaryotic and eukaryotic antimicrobial peptides, and the prediction algorithm is based on four models: support vector machines (SVM), random forests (RF), artificial neural network (ANN) and discriminant analysis (DA). The RF, SVM, and DA give a probability score (0 to 1) for the prediction. Higher probability indicates greater possibility of the peptide being antimicrobial. If the sequence is predicted to be antimicrobial or not antimicrobial, the results of ANN analysis are denoted as AMP or NAMP, respectively. The accuracy of the prediction results for the models is within the range of 87–93%.[30] The AMPA algorithm uses an antimicrobial propensity scale to generate an antimicrobial profile by means of a sliding window system. The propensity scale was derived using high-throughput screening results from the AMP Bactenecin 2A, a 12-residue peptide for which antimicrobial IC50 values for all amino acid replacements at each position are known (range 0.106–0.479). The calculated antimicrobial index deduced from the corresponding IC50 value for each amino acid substitution provides a good reference for the assessment of the protein sequence determinant. Low propensity value represents the most favored for antimicrobial peptide.[27] The APD3 program predicts the potential of an amino acid sequence to have antimicrobial activity by analyzing each amino acid residue and comparing the physicochemical properties of the sequence with those of the natural AMPs already deposited in the APD3 database.[28] The AMPs have a tendency to aggregate at the site of interaction with the bacterial cell membrane. The AGGRESCAN program calculates the aggregation-propensity values per amino acid (aaAV) derived previously from experimental data.[29]

### Binding of SPA4 peptide to bacteria and effect on bacterial growth

We examined whether SPA4 peptide directly binds to live bacteria and affects the extracellular growth of bacteria.[31] Bacterial cells harvested from 4 h mid-logarithmic growth phase were washed and incubated with 10, 50, 75, and 100 μM of FITC-SPA4 peptide in endotoxin-free buffer containing 1 mM CaCl_2_, 1 mM MgCl_2_, and 1 mM HEPES. The reaction mix was incubated at 37°C on a shaking water bath (85 rpm) for 45 min, and was swirled after every 15 min. Bacterial cells were washed three times with Dulbecco’s phosphate buffered saline (DPBS) by centrifugation at 6,000 rpm to remove free FITC-SPA4 peptide, and were then run on a flow cytometer. Polymyxin B is a cyclic cationic peptide antibiotic that binds to Gram-negative bacterial LPS.[32] The Oregon Green 514-conjugated (OG)-polymyxin B (Life Technologies, Grand Island, NY) was included as a positive control for binding to the bacteria. Oregon Green 514 and FITC are derivatives of fluorescein, and their fluorescence spectra demonstrate only moderate shift. The FL1 channel was used for flow cytometry analysis with both the fluorochromes (BD Biosciences, San Jose, CA). In addition, bacterial suspensions treated with FITC-SPA4 peptide or OG-polymyxin B were air-dried on glass slides and mounted with Vectashield (Vector Laboratories, Burlingame, CA). The emission capture was set at 493–523 nm for FITC and 519–646 nm for Oregon Green dye in the confocal microscope. All images were acquired with a 63X oil immersion objective in a Zeiss confocal microscope, and were processed using the Zeiss ZEN 2011 program. Bacteria alone, without any treatment with SPA4 peptide or polymyxin B, served as a negative control.

We assessed direct antibacterial activity of SPA4 peptide, as previously described.[33] An aliquot of mid-logarithmic culture of *P*. *aeruginosa* was pelleted at 5,000 X g at 4°C, and was washed with sterile DPBS. Diluted bacterial suspension was added to the wells of a Honeycomb 2 plate (Oy Growth Curves Ab, Ltd., Helsinki, Finland), which contained 1, 10, or 100 μg/ml SPA4 peptide, or an equivalent amount of vehicle. Absorbance readings (OD_600_) were taken at 37°C every 15 min for 17 h on the BioScreen C. After 17 h of incubation, an aliquot of the culture was serially diluted in DPBS, plated on agar plates, and incubated overnight at 37°C. Bacterial colonies were counted to obtain CFU/ml.

### Phagocytosis assay

The JAWS II dendritic cells were suspended in endotoxin-free phagocytosis assay buffer containing 1 mM CaCl_2_, 1 mM MgCl_2_, and 1 mM HEPES. The cell viability (90–95%), size, and volume were maintained in the phagocytosis assay buffer during the assay, as evidenced by the trypan blue staining and flow cytometric forward and side scatter pattern, respectively. One million JAWS II dendritic cells were incubated with GFP-expressing *P*. *aeruginosa* 8830 at different multiplicities of infection (MOI; cell to bacteria ratio), with or without 1% normal mouse serum for optimization. Different amounts of SPA4 peptide were added to the cell and bacterial mix. The reaction mix was incubated at 37°C for 45 min while shaking, and was swirled after every 15 min. Cells were washed three times with DPBS to remove free bacteria, and run on an Accuri flow cytometer (BD Biosciences, San Jose, CA). Cytochalasin D (Sigma-Aldrich, St. Louis, MO) served as a negative control. The cells and bacteria alone provided additional controls for setting the gates. Any shift in the FL1 (green) fluorescence of the gated cells was considered for bacterial phagocytosis.

### Transient transfection of JAWS II dendritic cells with wild-type TLR4 plasmid DNA

The JAWS II dendritic cells (1 x 10^6^ cells per well) were transfected with pDisplay control vector or the plasmid construct encoding wild-type mouse TLR4, per the published method.[22] Transiently transfected cells were washed and seeded in OptiMEM medium with reduced serum at a density of 10^5^ cells per well (Life Technologies, Grand Island, NY). First, we assessed whether transfected cells expressed functional TLR4. Briefly, we co-transfected JAWS II dendritic cells with plasmid constructs encoding wild-type mouse TLR4 or vector and NF-κB luciferase reporter plasmid DNAs. Cells were then challenged with LPS (100 ng/ml) for 5 h. Luciferase reporter activity was measured to assess TLR4-LPS-induced NF-κB activation. Arbitrary luminescence units were normalized with the total cellular protein. The TLR4 expression level was determined by dot immunoblotting using TLR4-specific antibody (Abcam, MA), per our published method.[34, 35]

### Labeling of *P*. *aeruginosa* with pHrodo Red, succinimidyl ester dye (pHrodo)

The pHrodo dye fluoresces at acidic pH. The pHrodo Red dye conjugates are non-fluorescent outside the cells, but fluoresce bright red in phagosomes.[14] *P*. *aeruginosa* PAO1 bacteria were conjugated to pHrodo dye (Molecular Probes-Life Technologies, Grand Island, NY), per the manufacturer’s instructions. *P*. *aeruginosa* was heat-killed at 65°C for 10 min, and was washed twice with DPBS. The bacterial suspension was then incubated with 0.93 mM pHrodo dye for 45 min at room temperature in the dark. The pHrodo-labeled bacterial pellet was washed and sonicated at room temperature.

### Localization of pHrodo-conjugated bacteria in the intracellular acidic phagolysosomes

The pHrodo-conjugated *P*. *aeruginosa* (one JAWS II dendritic cell to 320–780 bacteria) were added to the nontransfected or transfected JAWS II dendritic cells and primary mouse alveolar macrophages. After 1.5 h of incubation, the cells were treated with 75 μM SPA4 peptide or an equivalent amount of vehicle. Fluorescence readings were taken at 3.5 h of incubation at 530 nm excitation and 590 nm emission wavelengths, using the Synergy 2 multi-mode microplate reader (Biotek, Winooski, VT). The percent localization of pHrodo-conjugated bacteria into acidic phagolysosomes was calculated per the manufacturer’s instructions. After taking the fluorometric readings, we collected the cell-free supernatants and stored them at −80°C for further analysis. The cellular protein content was measured using the bicinchoninic acid assay (BCA; Pierce Biotechnology, Rockford, IL). The fluorometric readings were normalized with the amount of total cellular protein.

### Measurement of intracellular pH

We determined the effect of SPA4 peptide on the intracellular pH in unchallenged or *P*. *aeruginosa*-challenged cells using a pHrodo AM kit (Molecular Probes-Life Technologies, Grand Island, NY). *P*. *aeruginosa* PAO1 bacteria were prepared as described above for the pHrodo dye conjugation and phagocytosis. The intracellular pH was included to test whether SPA4 peptide itself causes any change in intracellular pH. Briefly, 100,000 cells per well were seeded in OptiMEM medium. After 1.5 h of incubation with *P*. *aeruginosa* PAO1, the cells were treated with 75 μM SPA4 peptide or vehicle, and 5 μM pHrodo Red AM dye. The standard buffer solutions (pH 4.5, 5.5, 6.5, and 7.5; provided with the kit) were mixed with 5 μM valinomycin, 5 μM nigericin, and pHrodo Red AM dye, and were added to the cells to plot a standard curve. Fluorescence readings were taken at 3.5 h of incubation at 530 nm excitation and 590 nm emission wavelengths.

### Confocal microscopy

After the flow cytometric or fluorometric readings were gathered, representative samples of cells were fixed in 3.5% paraformaldehyde solution for 20 min on ice, washed, and stained with the Hoechst 33342 dye (1 μg/ml), for nuclear staining. The slides were mounted with Vectashield (Vector Laboratories Inc., Burlingame, CA) and examined using confocal microscopy.

To confirm localization of bacteria within LAMP1-expressing phagolysosomes, representative fixed cells were permeabilized with 0.05% saponin solution and stained with Alexa Fluor 488-conjugated anti-mouse LAMP1 (expressed on lysosomal structures in cells) antibody (25 μg/ml; Biolegend, San Diego, CA).[36] Hoechst 33342 dye (1 mg/ml) was then added for nuclear staining. All images were acquired using a 63X oil immersion objective in a Zeiss confocal microscope, and were processed using the Zeiss ZEN 2011 program.

### Evaluation of SPA4 peptide in a mouse model of *P*. *aeruginosa* lung infection

Mice were anesthetized, positioned on the intubation platform, and intratracheally challenged with 1 x 10^7^ CFU (per OD_600_) of bacteria, using a gel-loading pipette tip and a laryngoscope (Penn-Century, Wyndmoor, PA). After 1 h of bacterial challenge, 20.86 nmol (50 μg) of SPA4 peptide or an equivalent amount of vehicle (endotoxin-free water) was administered intratracheally. Symptoms of sickness, including reactivity when held from tail, were scored on a scale of 0–3 and recorded as described earlier.[37] Mice were sacrificed after 5 h of bacterial challenge. Whole lung or lung tissue pieces were aseptically collected at the time of necropsy. The lungs were weighed, homogenized, plated on agar plates, and incubated at 37°C. The lung tissue homogenates were analyzed for levels of lactate as a marker of lung injury, activation status of intracellular signaling molecules, and secreted levels of inflammatory cytokines and chemokines. The levels of lactate were measured in diluted lung tissue homogenates using a fluorometric assay kit per the manufacturer’s instructions (BioVision, Milpitas, CA). The levels of cytokines (TNF-α, IL-1β, IL-6, IL-10, and IL-12p70) and chemokines (MCP-1, MIP-2, and KC) were measured in cell-free supernatants or lung tissue homogenates using ELISA. The amounts of TNF-α and IL-6 were measured per the method described earlier.[38] Commercially available ELISA kits were used for measuring the levels of IL-1β, IL-10, and IL-12p70 (Biolegend, San Diego, CA) and MCP-1, MIP-2, and KC (RnD Systems, Minneapolis, MN), per the manufacturers’ instructions. To obtain dry weights, the lungs were dried at 60°C in a vacuum oven for 48 h and weighed. The lung wet/dry weight ratios were compared between the groups. The lung tissue pieces were fixed in 10% formalin, sectioned, and stained with hematoxylin and eosin for examining the extent of tissue damage and inflammatory cells.

### Immunoblotting for total and phosphorylated (phospho) NF-κB-p65, and mitogen-activated protein kinases (MAPKs): p44/p42 ERKs, SAPK/JNK, and p38 MAPK

Forty μg of lung tissue homogenate proteins were separated on 4–20% Tris-glycine gel (Invitrogen, CA) and were immunoblotted with 1:1,000 diluted total or phospho-p44/42 MAPK (Erk1) (Tyr204)/(Erk2) (Tyr187), phospho-SAPK/JNK (Thr183/Tyr185), phospho-p38 MAPK (Thr180/Tyr182), or phospho-NF-κB-p65 (Ser536) antibody per the method published earlier.[20] The antibodies to the respective proteins and β actin were obtained from Cell Signaling Technology, Danvers, MA, and Santa Cruz Biotechnology, Dallas, TX. The densitometric units of immunoreactive bands were normalized with those of β actin.

### Statistics

Statistical significance was analyzed using the Student's or Mann Whitney *t*-test, or ANOVA, followed by Tukey’s post hoc analysis for multiple comparisons (Prism software, GraphPad, La Jolla, CA). Statistical significance was noted at the *p-*value of 0.05, or as otherwise indicated.

## Results

The amino acid sequence of the SPA4 peptide (GDFRYSDGTPVNYTNWYRGE) is derived from the C-terminal TLR4-interacting region of human SP-A.[18] We solved the structure of the SPA4 peptide by nuclear magnetic resonance spectroscopy, and studied the physicochemical features, which demonstrated its structural adaptability for binding to TLR4.[39]

### SPA4 peptide does not contain an antimicrobial domain. It neither directly binds to bacteria nor affects bacterial growth

The physicochemical features and blast search for antimicrobial regions within the amino acid sequence of SPA4 peptide indicated that it does not contain an antimicrobial domain (Table 1). Upon sequence alignment, the amino acid sequence of SPA4 peptide demonstrated only about 31.8–34.8% homology with five antimicrobial peptides (Formaecin 2, Odoraanain-V1, PP13, Andersonin-X1, and Formaecin 1 of Red Bulldog ant, wasp, and frog species) listed in the APD3 database (Table 1).

**Table 1:** Prediction of the potential antimicrobial domain in SPA4 peptide sequence based on physicochemical properties and algorithm models.

**Physicochemical properties**	
Molecular weight	2397.5
Molecular formula	C107H148N27O35S0
Net charge	-1 (at pH 7.4, cytoplasm), -1.1 (at pH 8.0, mitochondria), -0.9 (at pH 5.5, lysosome)[58]
pI	4.4[58]
Total hydrophobic ratio	15%
Grand average hydropathy value	-1.465
Wimley-White whole-residue hydrophobicity	2.1 kcal/mol
Molar extinction coefficient	10020
Protein-binding potential (Boman Index)	3.2 kcal/mol
a3vSA	-0.243
Number of aggregation hot spot regions	0
**Algorithm models**	
CAMPR3 models	SVM (0.049, NAMP); RF (0.079, NAMP); ANN (NAMP); DA (0.049, NAMP)
AMPA	The SPA4 peptide has no bactericidal stretch, and has a mean antimicrobial value of 0.249.
APD3: sequence alignment with the antimicrobial peptides in the database	Formaecin 2 (amino acid sequence: GRPNPVNTKPTPYPRL, similarity 34.78%); Odorranain-V1 (amino acid sequence: GLLSGTSVRGSI, similarity 33.33%); PP13 (amino acid sequence: GAARKSIRLHRLYTWKATIYTR, similarity: 32%); Andersonin-X1 (amino acid sequence: GLFSKFAGKGIVNFLIEGVE, similarity 31.81%); Formaecin 1 (amino acid sequence: GRPNPVNNKPTPHPRL, similarity 31.81%)

a3vSA, Aggregation-propensity value per amino acid sequence average; CAMPR3, Collection of anti-microbial peptides database; SVM, Support vector machines; RF, Random forests; ANN, Artificial neural network; DA, Discriminant analysis; NAMP, Not antimicrobial peptide; AMP, Antimicrobial peptide; AMPA, Antimicrobial sequence scanning system; APD3, Antimicrobial database

**Fig 1:**
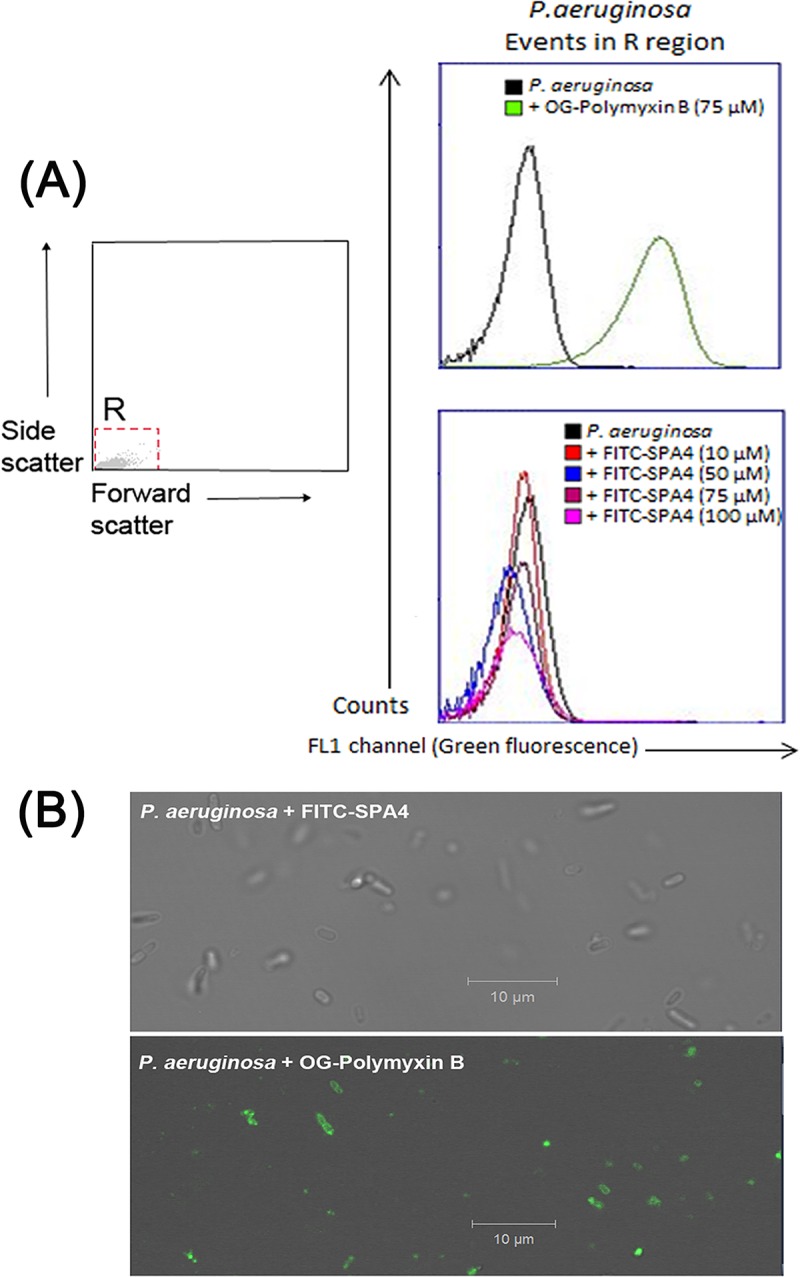
SPA4 peptide does not bind to live *P*. *aeruginosa*. Live non-GFP *P*. *aeruginosa* bacteria were incubated with 10, 50, 75, and 100 μM of FITC-SPA4 peptide, or 75 μM of Oregon Green (OG)-polymyxin B (positive control). No shift was observed in flow cytometric histograms of *P*. *aeruginosa* in the FL1 channel when incubated with FITC-SPA4 peptide. In contrast, a significant shift was observed in flow cytometric histograms of bacteria in the FL1 channel when incubated with OG-polymyxin B, which binds to bacteria **(A)**. Confocal microscopic images of live non-GFP bacteria incubated with FITC-SPA4 peptide (75 μM) and OG-polymyxin B (75 μM). The confocal images were obtained using brightfield and FL1 channels. Absence of fluorescence indicates no binding of FITC-SPA4 peptide to the bacteria. Green fluorescence indicates binding of OG-polymyxin B to the bacteria **(B)**.

Surfactant protein-A has been shown to bind to and directly kill bacterial pathogens.[4, 7] Direct binding of the FITC-SPA4 peptide to live bacterial cells was assessed using flow cytometry and confocal microscopy. Polymyxin B strongly binds to LPS in bacterial cell walls. Incubation of bacterial cells with OG-polymyxin B caused a significant shift in the fluorescence peak in flow cytometric histograms, indicating binding (Fig 1A). Bright green fluorescence of polymyxin B-bound bacteria was visible in confocal images (Fig 1B). However, we observed no binding of FITC-SPA4 peptide to live *P*. *aeruginosa*.

**Fig 2:**
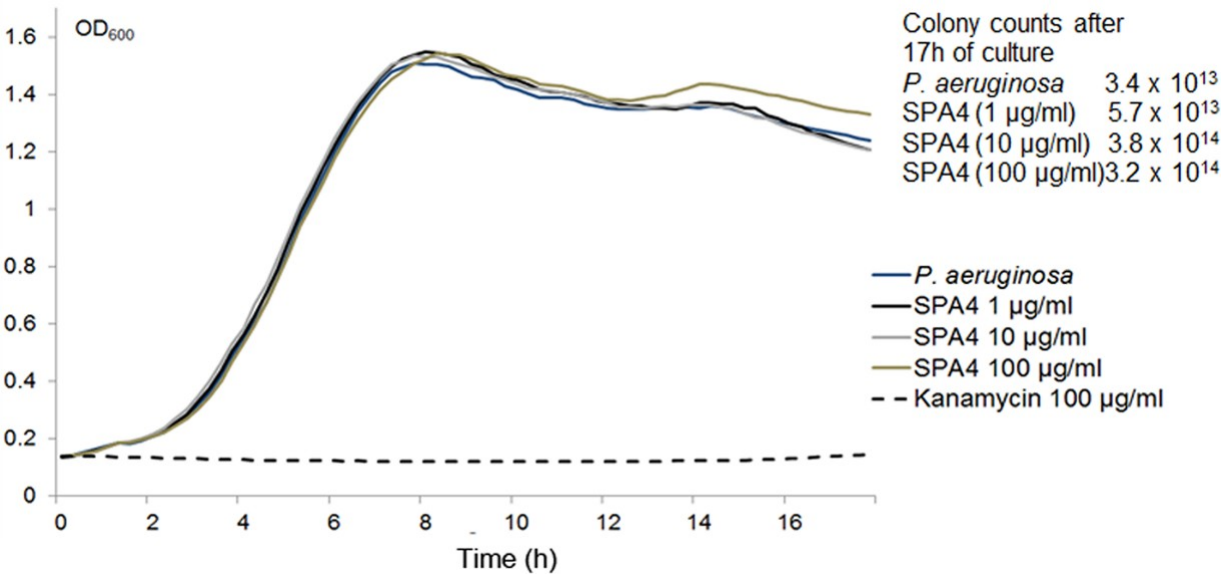
SPA4 peptide does not affect the growth of *P*. *aeruginosa*. Growth curves (OD_600_ versus time in hours) of *P*. *aeruginosa* cultured over 17 h in the presence of increasing concentrations of SPA4 peptide or vehicle. Kanamycin (100 μg/ml) was used as a positive control for growth inhibition. Colony counts (CFU/ml) obtained at 17 h are included within the figure.

We also measured the effect of SPA4 peptide on the growth of *P*. *aeruginosa*. Mid-log phase bacteria were cultured in liquid medium in the presence of SPA4 peptide or vehicle. Addition of SPA4 peptide did not affect bacterial growth, as assessed by OD_600_ or colony counts (Fig 2).

### SPA4 peptide enhances phagocytic uptake and localization of *P*. *aeruginosa* into phagolysosomes, and suppresses the release of TNF-α

**Fig 3:**
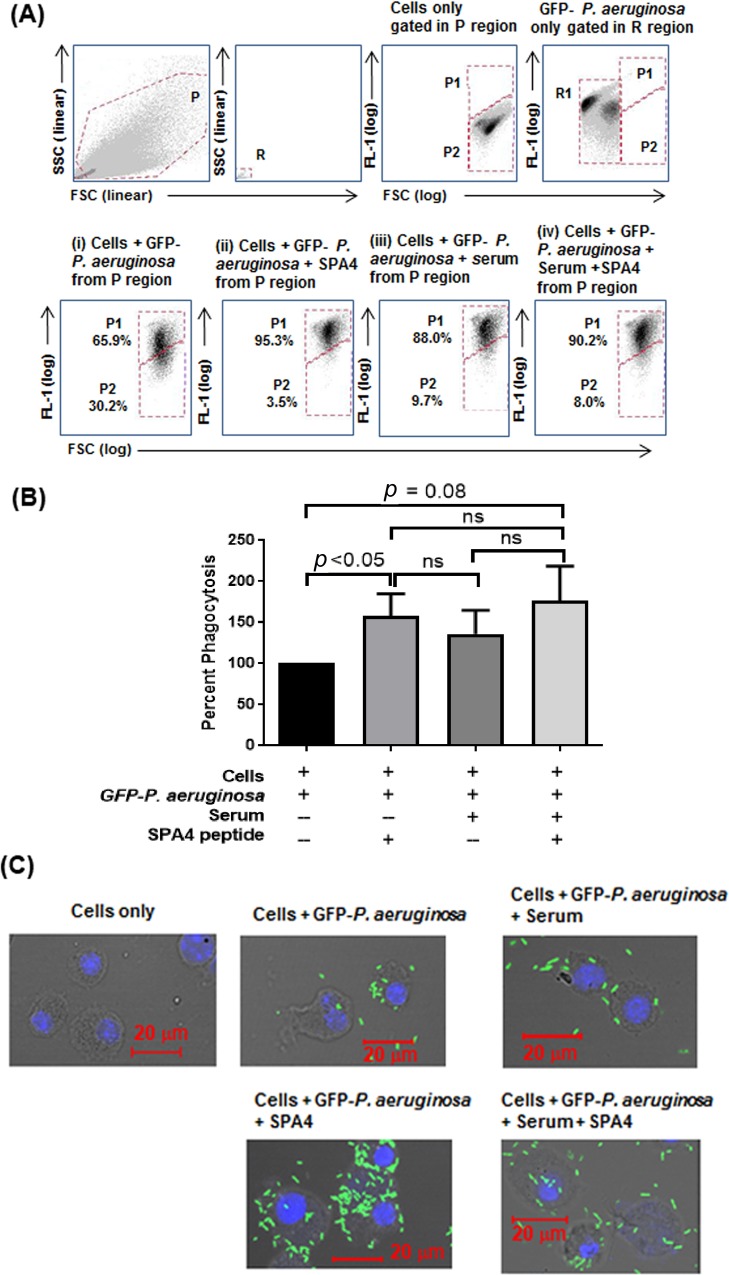
SPA4 peptide induces phagocytosis of live GFP-*P*. *aeruginosa*. Flow cytometric FSC versus SSC dot plots of JAWS II dendritic cells (gated in region P) and bacteria (gated in region R). The FSC versus FL1 dot plots show free GFP-*P*. *aeruginosa* in the R1 region (negative control) and cells only (negative control) in P2 region. A shift is noted when the cells take up the GFP-*P*. *aeruginosa*. The flow cytometric dot plot charts demonstrate the cells with phagocytosed bacteria in P1 area. Numbers indicate percent of cells with phagocytosed GFP-*P*. *aeruginosa* (in P1) or without any bacteria (in P2). Results shown here are from one representative experiment **(A)**. The bar chart shows mean (+ *SEM*) of percent phagocytosis of GFP-*P*. *aeruginosa* as compared with one hundred percent basal phagocytosis from eight experiments, respectively. The *p*-values determined by *t*-test are shown within the figure **(B)**. Representative confocal micrographs of cells alone or with phagocytosed GFP-*P*. *aeruginosa*. Images taken at brightfield and fluorescence channels were superimposed. Green fluorescence is of GFP-*P*. *aeruginosa*. Blue staining is of cell nuclei **(C)**.

TLR4 plays a critical role in pathogen-recognition, phagocytosis, and inflammation.[40] Thus, we investigated the effect of SPA4 peptide on phagocytic uptake and localization of bacteria in the intracellular acidic phagolysosomes. For measurement of phagocytic uptake, the JAWS II dendritic cells were incubated with live GFP-*P*. *aeruginosa* in the presence or absence of SPA4 peptide, washed, and assessed for a shift in cell-associated green fluorescence by flow cytometry. First, we confirmed the stability of GFP expression of the bacteria under the phagocytosis assay conditions. A shift in the flow cytometric histogram of live bacteria in the FL1 channel and green fluorescent colonies confirmed GFP expression by GFP-*P*. *aeruginosa* (Fig 3A). At the same time, we noted the flow cytometric histogram of the JAWS II dendritic cells used in the assay in the FL-1 channel for each experiment. The histogram plots of GFP-*P*. *aeruginosa* and JAWS II dendritic cells were well-separated in the FL-1 channel, and were stable over time (Fig 3A). Therefore, any shift in the histogram plot of JAWS II dendritic cells incubated with GFP-*P*. *aeruginosa* indicated bacterial uptake.

Phagocytosis was determined after incubation of cells with GFP-*P*. *aeruginosa* for 45 minutes. In the initial experiments, the multiplicities of infection (cell-to-bacteria ratios) and SPA4 peptide concentrations were used at 1:25–1:100 and 10–75 μM, respectively. The MOI of 1:100 and SPA4 peptide concentration of 75 μM were used for comprehensive experiments in the absence and presence of 1% normal mouse serum. Results were analyzed by flow cytometry. The cell debris was gated out, and all the cells in P region were included. Only the events in the R (based on forward: FSC, versus side: SSC scatter pattern) or R1 (based on FSC versus FL1) regions were gated out for any green fluorescence, due to free bacteria. Experiments under these conditions consistently demonstrated that the SPA4 peptide induced bacterial phagocytosis by JAWS II dendritic cells (Fig 3A and 3B). Results were confirmed by visualization of representative samples for intracellular phagocytosed bacteria using confocal microscopy. A Z-stack of cells was captured. The percentages of cells with internalized GFP-bacteria (phagocytosed) were noted at the specific plane of the Z-stack when the cell nucleus was visible. Analysis using confocal microscopy showed an increase in the percentage of cells with phagocytosed bacteria after SPA4 peptide treatment; these results were consistent with the flow cytometry analysis. We observed very few bacteria towards the outer edge of the cells; these bacteria could still be tightly attached to the cell (Fig 3C).

**Fig 4:**
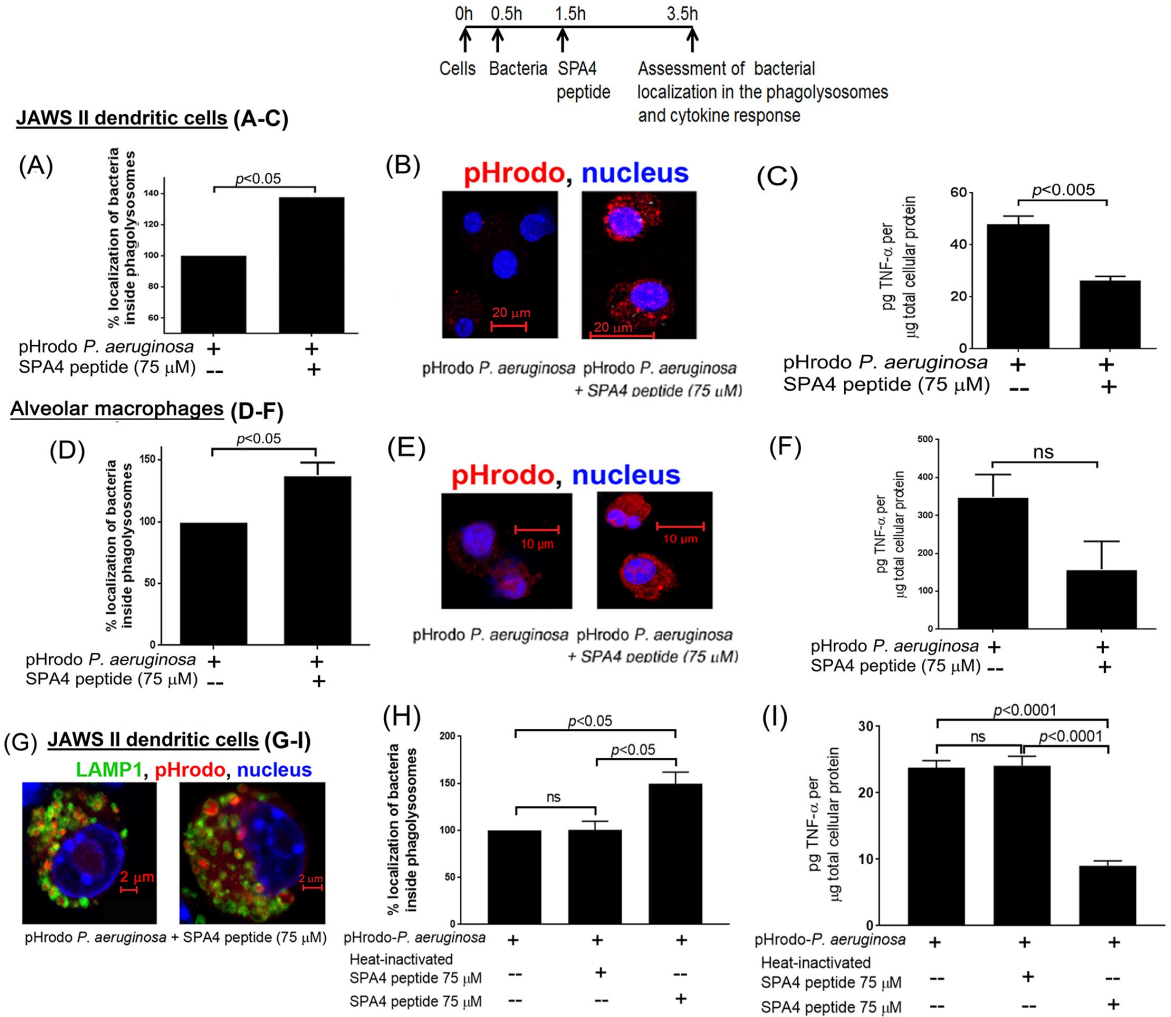
SPA4 peptide treatment induces localization of bacteria inside acidic phagolysosomes of JAWS II dendritic cells and alveolar macrophages, but suppresses the TNF-α response. The flow chart depicts the timing of adding the bacteria and SPA4 peptide, and of making assessments. Percent localization of pHrodo-labeled *P*. *aeruginosa* in acidic phagolysosomes of JAWS II dendritic cells **(A)** and alveolar macrophages **(D)** after treatment with SPA4 peptide. Confocal images show localization of red fluorescent pHrodo-labeled *P*. *aeruginosa* inside the acidic phagolysosomes of JAWS II dendritic cells **(B)** and alveolar macrophages **(E)**. Secreted levels of TNF-α cytokine in cell-free supernatants of JAWS II dendritic cells **(C)** and alveolar macrophages **(F)** exposed to pHrodo-labeled *P*. *aeruginosa*. The cell nucleus stained with Hoechst 33342 dye is shown in blue. LAMP1 staining (in green) confirms the localization of pHrodo-labeled bacteria inside the LAMP1-expressing phagolysosome **(G)**. Percent localization of pHrodo-labeled P. aeruginosa in phagolysosomes **(H)** and TNF-α in cell-free supernatants **(I)** of cells treated with heat-inactivated SPA4 peptide. The *p*-values determined by *t*-test are shown within figures.

In a separate assay, we used *P*. *aeruginosa* bacteria labeled with pHrodo dye, which fluoresce only at an acidic pH and reveal localization within acidic compartments of the cells.[14] SPA4 peptide treatment enhanced localization of *P*. *aeruginosa* in the acidic compartments of JAWS II dendritic cells and primary alveolar macrophages by 40%, compared with basal phagocytosis (Fig 4A, 4B, 4D and 4E). Localization of bacteria (red fluorescence) inside the lysosomal-associated membrane protein 1 (LAMP1)-expressing phagolysosomes was confirmed using confocal microscopy (Fig 4G). The SPA4 peptide treatment did not affect the intracellular pH of unchallenged cells [7.3 ± 0.23 (mean ± standard error of measurement) in SPA4 peptide-treated *versus* 7.4 ± 0.15 in vehicle-treated cells]. However, SPA4 peptide treatment slightly induced the intracellular pH of *P*. *aeruginosa*-challenged cells (5.7 ± 0.4 in SPA4 peptide-treated *versus* 4.0 ± 0.4 in vehicle-treated cells, *p*<0.05, ANOVA).

We also measured secreted levels of TNF-α in the supernatants of JAWS II dendritic cells and alveolar macrophages challenged with pHrodo-labeled *P*. *aeruginosa*. We found that the SPA4 peptide suppressed TNF-α levels after challenge with *P*. *aeruginosa* (Fig 4C and 4F). Heat-inactivation inhibited the SPA4 peptide activity (Fig 4H and 4I).

### Pro-phagocytic and anti-inflammatory activity of SPA4 peptide occurs through its interaction with TLR4

**Fig 5:**
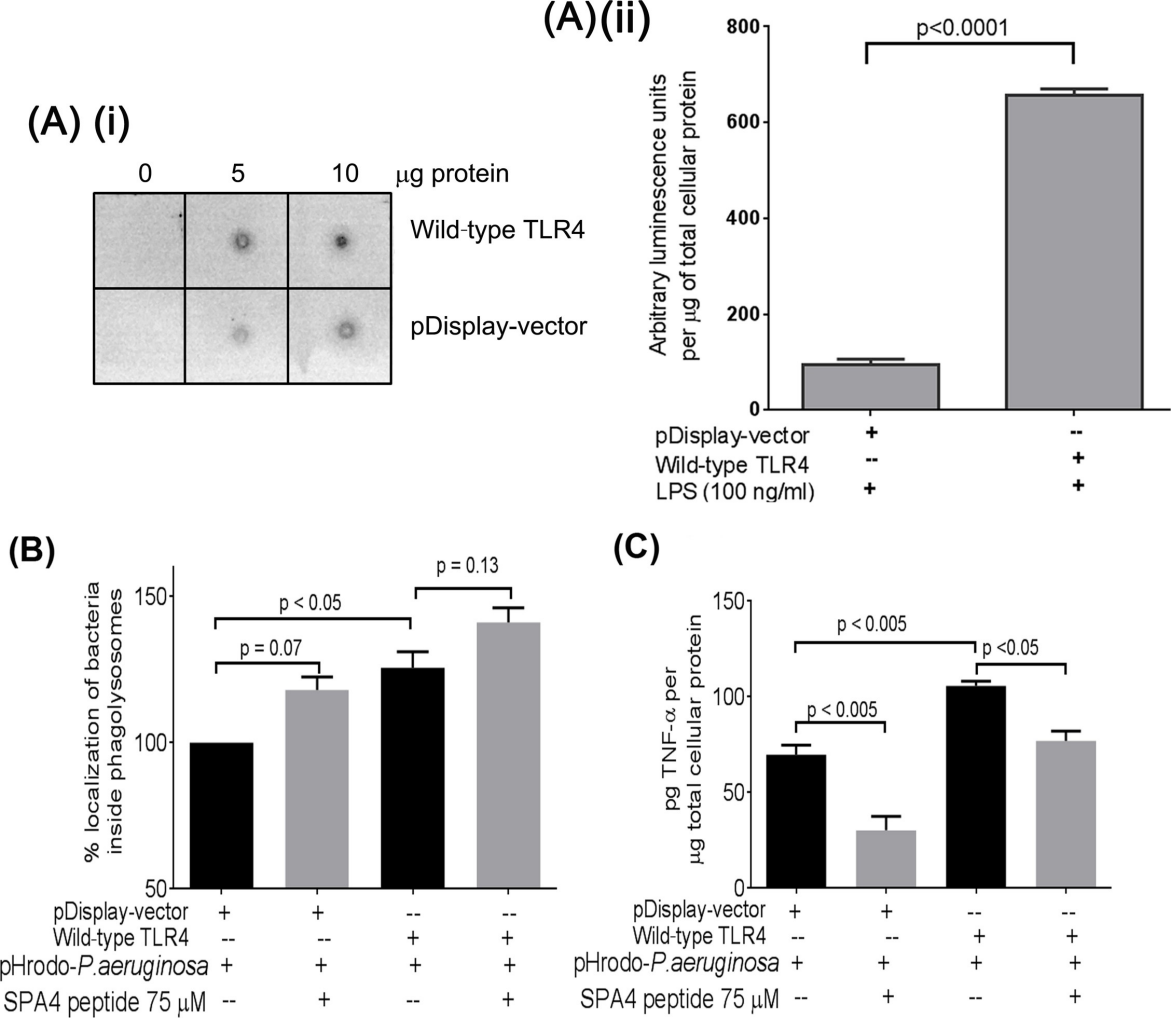
Effect of SPA4 peptide on localization of bacteria inside the acidic phagolysosomes and TNF-α levels in cell-free supernatants of JAWS II dendritic cells overexpressing TLR4. The cells were co-transfected with NF-κB-luciferase reporter plasmid construct and plasmid construct encoding wild-type TLR4 or vector control (pDisplay-vector). Dot immunoblotting results demonstrated overexpression of TLR4 in cells transfected with wild-type TLR4. Total cell lysate proteins (5 and 10 μg) were immunoblotted with TLR4-specific antibody **(A, i)**. Transfected cells were challenged with LPS (100 ng/ml) and arbitrary luminescence values for NF-κB-luciferase activity were normalized with total cellular protein **(A, iii)**. Cells transfected with plasmid construct encoding wild-type TLR4 or vector control (pDisplay-vector) were exposed to pHrodo-labeled *P*. *aeruginosa*, and treated with SPA4 peptide **(B, C)**. Red fluorescence due to internalized bacteria was quantified by fluorometry, and percent localization of bacteria was calculated relative to control; bars represent the mean + *SEM* of results from three separate experiments **(B)**. TNF-α levels were measured in cell-free supernatants using ELISA and were normalized with total cellular protein **(C)**. TNF-α results are from one representative experiment performed in triplicate. The *p*-values were determined by *t*-test **(A, iii)** or one-way ANOVA (Tukey’s post-hoc test) **(B, C)**.

It is well established that TLR4 stimulates phagocytosis and inflammatory response. To mimic the increased expression of TLR4, we transfected the JAWS II dendritic cells with wild-type TLR4. The cells transfected with wild-type TLR4 expressed increased levels of TLR4 compared with control (Fig 5A, i). As expected, transfection with TLR4 and NF-κB luciferase reporter plasmid DNAs increased the NF-κB reporter activity in response to *Escherichia coli*-LPS (100 ng/ml), compared with control vector-transfected cells (Fig 5A, ii). These results confirm that the JAWS II dendritic cells transfected with wild-type TLR4 overexpressed functionally active TLR4. Our results further demonstrate that the SPA4 peptide treatment increased the intracellular localization of bacteria in phagolysosomes in cells transfected with plasmid DNAs encoding vector. As expected, we observed an increased localization of bacteria in cells overexpressing TLR4. However, it was not significantly affected further when cells with overexpressed TLR4 were treated with SPA4 peptide (Fig 5B). The secreted levels of TNF-α were significantly reduced in cells transfected with plasmid DNAs encoding vector or TLR4 (Fig 5C).

### SPA4 peptide treatment reduces bacterial burden and inflammatory mediators in lung, and alleviates lung injury

**Fig 6:**
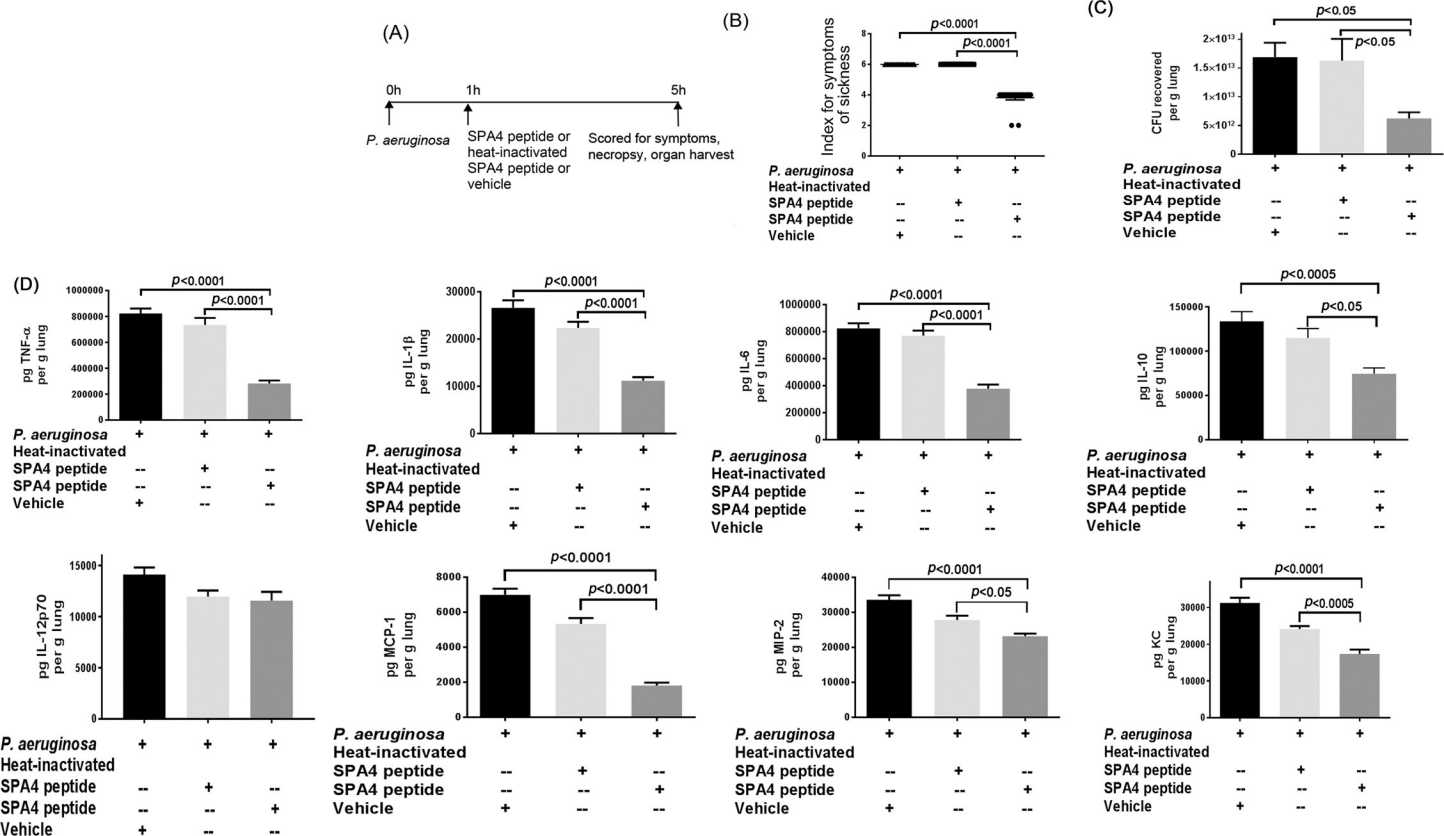
SPA4 peptide treatment improves host defense in a mouse model of *P*. *aeruginosa* lung infection. Flow chart depicts the schedule and dose of challenge with live *P*. *aeruginosa* and treatment with SPA4 peptide or heat-inactivated SPA4 peptide or vehicle *via* intratracheal route **(A)**. Mice were sacrificed at 5 h of bacterial challenge, and their lungs were harvested. Before euthanasia, the symptoms of sickness were scored in mice. Average indices for the symptoms of sickness are shown in **(B)**. The bar chart (Mean + *SEM*) shows recovered CFU of *P*. *aeruginosa* per g lung **(C)**. Levels of cytokines (TNF-α, IL-1β, IL-6, IL-10, and IL-12p70) and chemokines (MCP-1, MIP-2, and KC) (pg per g lung) were measured in lung tissue homogenates by ELISA **(D)**. Data are from 12 mice per group included in two separate experiments performed on different occasions. The *p*-values were determined by one-way ANOVA (Tukey’s post-hoc test).

We next tested whether the pro-phagocytic and anti-inflammatory activity of SPA4 peptide translates to an improvement of host defense *in vivo*. We challenged C57BL6/J mice with live *P*. *aeruginosa*. We previously reported that SPA4 peptide is effective when given therapeutically. Thus, in this short-term study, we treated the mice with SPA4 peptide after 1 h of infectious challenge (Fig 6A). We observed that mice treated with SPA4 peptide were more alert and reactive when held by the tail, as compared with those treated with vehicle (Fig 6B).[37] Treatment with SPA4 peptide significantly reduced the bacterial burden, cytokines (TNF-α, IL-1β, IL-6, IL-10, and IL-12), and chemokines (MCP-1, MIP-2, and KC) in lungs of infected mice after 5 h of infectious challenge (Fig 6C and 6D). The heat-inactivated SPA4 peptide was not effective in alleviating the symptoms or in reducing the bacterial burden and inflammatory parameters.

**Fig 7:**
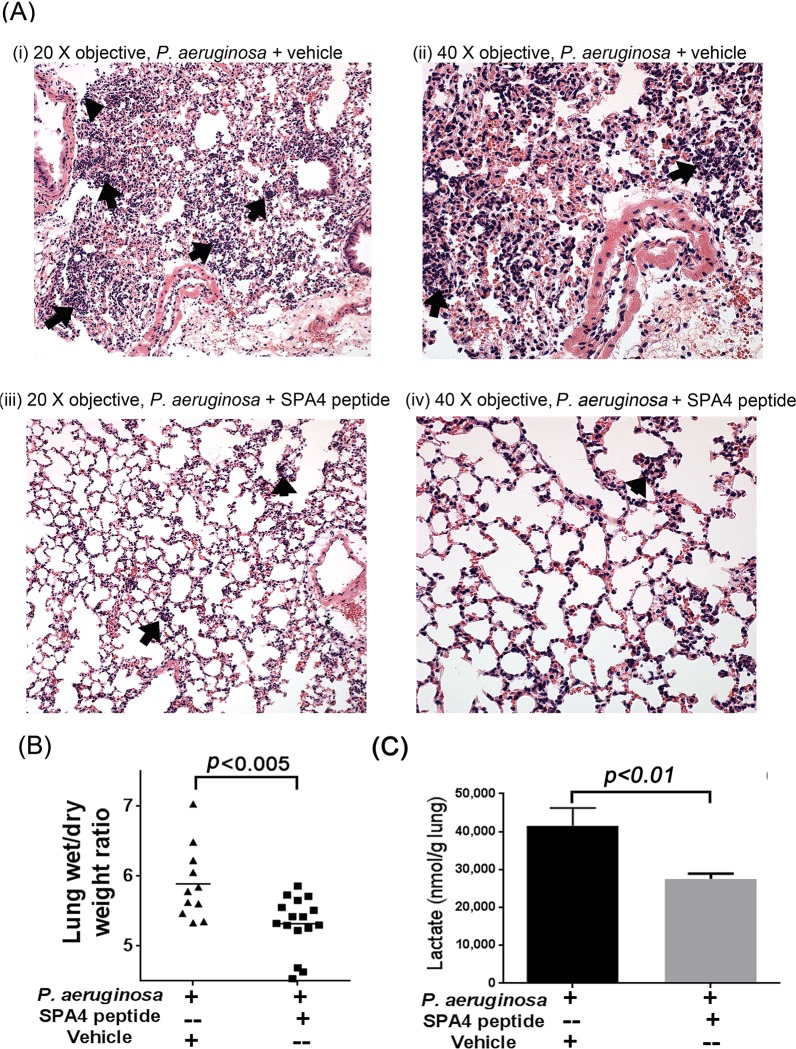
Representative micrographs of H & E-stained mouse lung tissues (with 20X and 40X objectives) harvested from P. aeruginosa-challenged and SPA4 peptide- or vehicle-treated mice **(A)**. The lungs appear to be markedly hypercellular in vehicle-treated, *P*. *aeruginosa*-infected mice due to an acute inflammatory cell response consisting mostly of neutrophilic leukocytes (arrows) **(A, i)**. Marked influx of the neutrophilic leukocytes (arrows) was multifocal to diffuse, with most cells present within the smaller vessels. Fewer cells appeared to be present within the interstitium, including the alveolar sacs **(A, ii)**. In the SPA4 peptide-treated mice, the lungs appeared to be only mildly cellular (arrows) **(A, iii)**. The mild influx of cells (arrows), consisting mostly of neutrophilic leukocytes, appears to have a predominantly intravascular location involving the smaller vessels. A few cells also appear to be located within the interstitium **(A, iv)**. Lung edema was determined by lung wet/dry weight ratios **(B)**. The lactate levels (pmol per g lung) were measured in lung tissue homogenates of *P*. *aeruginosa*-infected, SPA4 peptide- or vehicle-treated, mice **(C)**. Data are from 12 mice per group included in two separate experiments performed on different occasions. The *p*-values were determined by one-way ANOVA (Tukey’s post-hoc test).

Tissue sections of lung from SPA4 peptide-treated mice exhibited less tissue damage and fewer foci of inflammatory cells compared with those from *P*. *aeruginosa-*infected, vehicle-treated, control mice (Fig 7A). SPA4 peptide treatment also reduced lung edema in infected mice, as evidenced by reduced lung wet/dry weight ratios compared with vehicle-treated controls (Fig 7B). An increased level of lactate relates to tissue hypoxia and injury, and has been used as a biomarker of tissue injury.[41–43] The SPA4 peptide treatment significantly reduced the levels of lactate in mouse lungs (Fig 7C).

### SPA4 peptide treatment suppresses the phosphorylation of p54 SAPK/JNK, p38 MAPK, and NF-κB-p65 in lung tissues of *P*. *aeruginosa*-infected mice

**Fig 8:**
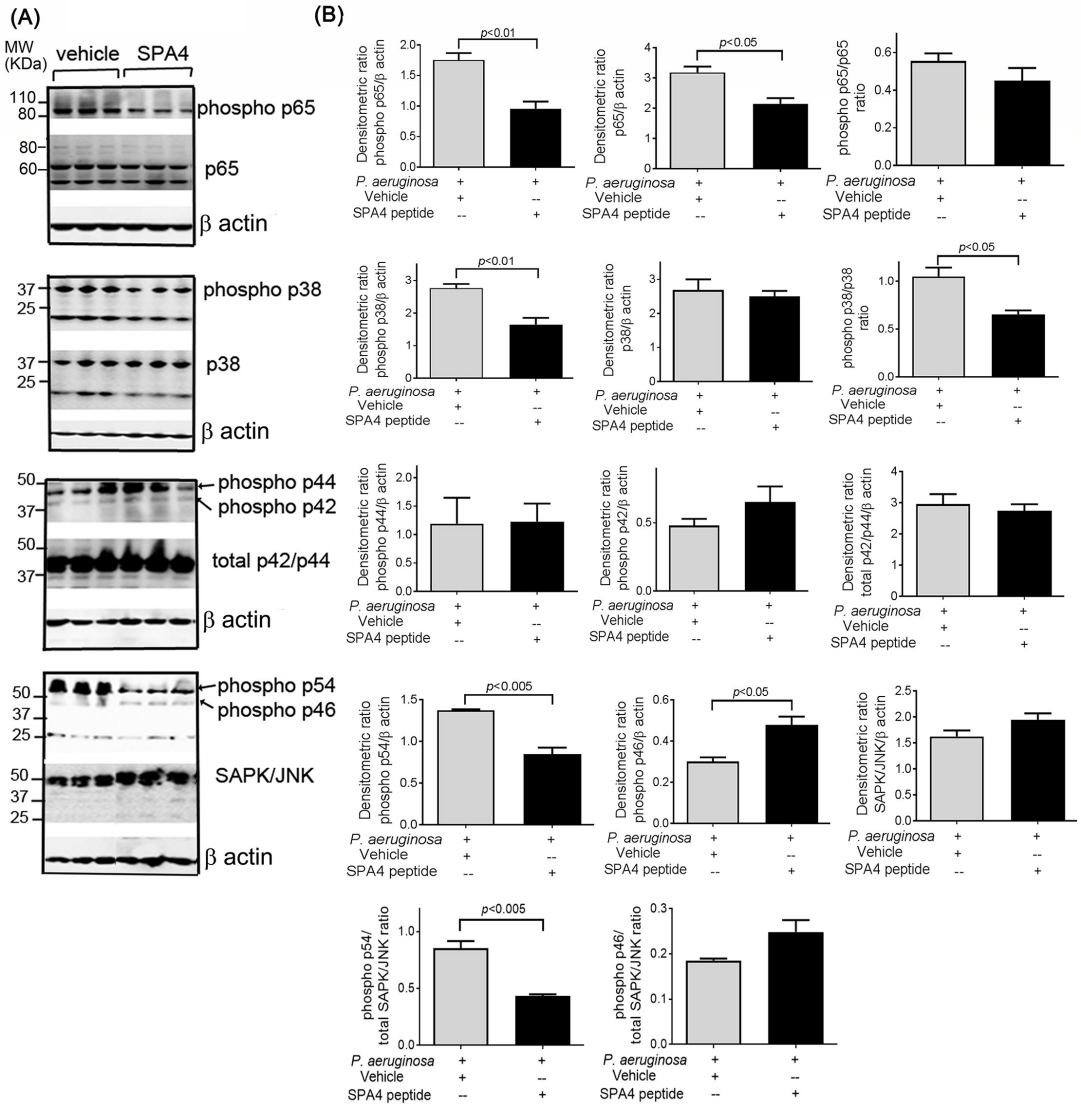
Expression of total and phosphorylated (phospho) p42/p44 MAPK, SAPK/JNK, p38 MAPK, and NF-κB p65 in lung tissues. Forty μg of lung tissue homogenate protein from *P*. *aeruginosa*-infected and SPA4 peptide- or vehicle-treated mice were separated on SDS-PAGE gel and immunoblotted for phospho NF-κB-p65 (phospho p65), NF-κB-p65 (p65), phospho p38 MAPK (phospho p38), p38 MAPK (p38), phospho p44/p42 MAPK (phospho p44 and phospho p42), total p42/p44 MAPK (total p42/p44), phospho SAPK/JNK (phospho p54 and phospho p46), and SAPK/JNK. The immunoblots were stripped and re-probed with β actin for protein loading. The immunoblots shown are from lung tissue homogenates of three mice in each group, representative of immunoblots performed in separate samples from 12 mice per group in two experiments **(A)**. The densitometric units of immunoreactive bands for NF-κB and MAPK proteins in **(A)** were normalized with those of β actin. Average densitometric ratios are shown as mean + *SEM* **(B)**. Ratios of phospho p65/p65, phospho p38/p38, phospho p54/total SAPK/JNK, and phospho p46/total SAPK/JNK are also shown. The *p*-values were determined by *t*-test.

Earlier, we published that the SPA4 peptide affects the TLR4-MYD88-dependent activation of NF-κB and release of TNF-α against LPS stimuli.[19, 20] The TLR4-MYD88-dependent signaling also stimulates MAPK, which contributes to the inflammation process.[44, 45] Our results demonstrate that SPA4 peptide treatment reduces total and phosphorylated (phospho) NF-κB-p65, phospho p38 MAPK, and phospho p54 SAPK/JNK in lung tissue homogenates of *P*. *aeruginosa*-infected mice, compared with vehicle-treated controls. SPA4 peptide had undetectable or moderate effects on total p38, total and phospho p42/p44 ERK, or total SAPK/JNK, and phospho p46 SAPK/JNK expression (Fig 8).

## Discussion

Using multidisciplinary approaches, we identified the SPA4 peptide as the lead peptide from TLR4-interacting regions of SP-A.[18, 20] Our results demonstrated that the SPA4 peptide binds to TLR4 and reduces LPS-MYD88-NF-κB activity, TLR4-priming of the inflammasome, and the inflammatory cytokine response.[18, 20, 22] In this report, we investigated the effect of SPA4 peptide on phagocytosis and clearance of *P*. *aeruginosa* and the associated inflammatory response in immune cells, and bacterial burden and inflammatory and lung injury parameters in a mouse model of *P*. *aeruginosa*-induced lung infection. All parameters were analyzed on a short-term basis specifically to delineate the effect of SPA4 peptide on early events of TLR4 signaling, which may be critical in orchestrating the long-term immunity, local homeostasis, and biological effect.

TLR4 recognizes and stimulates uptake of LPS-expressing Gram-negative bacteria, and regulates innate and adaptive immunity. Two main early immune functions are activated at the upstream level upon TLR4 complex formation: (i) uptake of the LPS or pathogen and receptor endocytosis, and (ii) induction of signal transduction. The recognition and uptake of LPS and live bacteria are orchestrated through an endocytosis motif present on the extracellular domain of the TLR4; the cytoplasmic tail of TLR4 is not required.[46, 47] The intracellular domain of TLR4 is involved in inducing the signaling cascade. The TIR domain in the cytoplasmic tail of TLR4 recruits adaptor proteins, which stimulate the MYD88 and TRIF signaling pathways. The MYD88-mediated signaling occurs at the plasma membrane and is involved in early immune response. The activation of TRIF pathway occurs in the endosomal compartment after internalization of the TLR4 complex. Eventually, the MYD88- and TRIF-signaling pathways induce the IκB kinase (IKK) complex, and MAPK family (ERK, JNK and p38), leading to activation of NF-κB and activator protein (AP)-1 transcription factors, respectively. These signaling molecules and regulators have been recognized for their role in TLR4-stimulated immune responses against infectious and inflammatory stimuli.[17, 48, 49]

Levine *et al*. reported the importance of SP-A in controlling infection by demonstrating increased susceptibility of SP-A-deficient mice to infection.[50] The SP-A exerts its host defense function against *P*. *aeruginosa* through its direct bactericidal activity [4, 5, 7] and enhanced phagocytosis.[51, 52] However, unlike SP-A, the SPA4 peptide did not directly bind to live bacterial cells (Fig 1) or LPS.[19] The SPA4 peptide also did not affect the binding of LPS to the host immune cells.[22] In this study, we observed that the SPA4 peptide induces bacterial uptake and intracellular localization of *P*. *aeruginosa* in phagolysosomes, yet reduces the inflammatory response. While the pro-phagocytic activity was maintained, the anti-inflammatory response was augmented in cells overexpressing TLR4 (Fig 5B and 5C). These results do not rule out the possibility of SPA4 peptide affecting other intracellular TLR4-dependent or independent mechanisms. The absence of antimicrobial domain in the SPA4 peptide (Table 1), and no detectable bacterial killing activity (Fig 2), suggest that SPA4 peptide exerts its host defense function against bacterial pathogens through immunomodulation. Similar results were observed with *E*. *coli* (unpublished results).

When SPA4 peptide was administered therapeutically in *P*. *aeruginosa*-infected mice, the pro-phagocytic and anti-inflammatory activity of SPA4 peptide led to reduced bacterial burden and decreased levels of cytokines and chemokines (Fig 6). These results corresponded with reduced levels of lactate (Fig 7C). It was noted that the heat-inactivated SPA4 peptide does not exert host defense function against *P*. *aeruginosa* (Fig 6). These results confirm the structure-activity relationship of SPA4 peptide as predicted in our previously published reports.[18, 39] Further analyses of molecular basis of SPA4 peptide structure, binding to TLR4, and immunomodulation will provide better understanding of its mechanism of action at cellular, organ, and whole organism levels.

It is established that TLR4-activation stimulates the signaling cascade, leading to phosphorylation of NF-κB p65, p38MAPK, and SAPK/JNK, which are downregulated in the lungs of *P*. *aeruginosa*-infected, SPA4 peptide-treated mice (Fig 8). While some aspects of SPA4 peptide activity on TLR4-induced inflammatory signaling have been addressed,[19, 20, 22] additional studies are warranted to further delineate the molecular mechanism of SPA4 peptide activity against live pathogens.

TLR4 has been identified as an attractive target for immunomodulation because of its increased expression on infiltrating immune cells and multifaceted roles during infection. Several TLR4 immunomodulators are currently being developed as a way to control inflammation, primarily during sepsis.[53, 54] Therapeutic applications of most studied TLR4 modulators remain incompletely explored.[54–57] Our results show that the SPA4 peptide has anti-inflammatory properties, but also exerts a pro-phagocytic response against Gram-negative bacteria (Figs 3–5). Based on the results presented here, the TLR4-interacting SPA4 peptide may offer a novel host-targeted approach for the treatment of antibiotic-resistant, Gram-negative bacterial infections that are accompanied by inflammation and tissue injury.

## Supporting information

S1 File The ARRIVE guidelines checklist. (PDF)
